# Transduction of rat and human adipose-tissue derived mesenchymal stromal cells by adeno-associated viral vector serotype DJ

**DOI:** 10.1242/bio.058461

**Published:** 2021-09-08

**Authors:** Ekaterina S. Zubkova, Irina B. Beloglazova, Elizaveta I. Ratner, Daniyar T. Dyikanov, Konstantin V. Dergilev, Mikhail Yu. Menshikov, Yelena V. Parfyonova

**Affiliations:** 1 National Medical Research Center of Cardiology, Moscow 121552, Russian Federation; 2 Department of Biochemistry and Molecular Medicine, Faculty of Medicine, Lomonosov Moscow State University, Moscow 119991, Russian Federation

**Keywords:** Adeno-associated viral vectors, Gene therapy, Mesenchymal stromal cells

## Abstract

*Ex vivo*, gene therapy is a powerful approach holding great promises for the treatment of both genetic and acquired diseases. Adeno-associated virus (AAV) vectors are a safe and efficient delivery system for modification of mesenchymal stem cells (MSC) that could maximize their therapeutic benefits. Assessment of MSC viability and functional activity after infection with new AAV serotypes is necessary, due to AAV tropism to specific cell types. We infected human and rat adipose-tissue MSC with hybrid AAV-DJ serotype vectors carrying GFP and SCF genes. GFP expression from AAV-DJ was about 1.5-fold superior to that observed with AAV-2 and lasted for at least 21 days as was evaluated by flow cytometry and fluorescence microscopy. AAV-DJ proves to be suitable for the infection of rat and human MSC with a similar efficiency. Infected MSC were still viable but showed a 25-30% growth-rate slowdown. Moreover, we found an increase of SERPINB2 mRNA expression in human MSC while expression of other oxidative stress markers and extracellular matrix proteins was not affected. These results suggest that there is a differential cellular response in MSC infected with AAV viral vectors, which should be taken into account as it can affect the expected outcome for the therapeutic application.

## Abstract (precis)

**Summary:** Adeno-associated viral vectors are widely used for gene delivery but their impact on the different cell types varies greatly and is not well understood. We describe effects of two most popular AAV serotypes on mesenchymal stromal cells of rat and human origin.

## INTRODUCTION

Multipotent mesenchymal stromal cells (MSC) have a great capacity for self-renewal and ability to secrete a broad range of bioactive molecules (Sagaradze et al., 2018). MSC can be readily isolated at sufficient quantity for usage in regenerative medicine. Besides, MSCs can be used as a vehicles for therapeutic gene delivery to the injured sites. They could significantly increase the safety and efficiency of gene therapy minimizing the immune response, elicited by viral vectors, due to their intrinsic immunosuppressive properties.

Recombinant adeno-associated viral vectors (AAV) demonstrate outstanding safety profile and does not cause any known human or animal diseases. Thus, they have been considered as one of the preferred vectors for use in clinical applications.

AAV possess several advantages including its ability to infect both dividing and quiescent cells and rarity of host genome integration that reduce the risks of insertional mutagenesis. AAV-mediated transgene expression is not permanent, but could persist over a period from several months to several years (Sehara et al., 2017) depending on the immune response and the turnover rate of the infected cells (Lakhan et al., 2015). Therefore, inactivation of the transgene may not be necessary after the disease is cured. And finally, AAV is significantly less immunogenic than adenoviral vectors (Jooss et al., 1998) but as AAV is widespread in nature, more than 70% of people are seropositive for one or several AAV serotypes (Grimm et al., 2008). New chimeric capsids were created on the basis of 12 known natural serotypes to further reduce immunogenicity. AAV-DJ serotype is a chimera of AAV2/8/9 serotypes and distinguished from its closest natural relative (AAV-2) by 60 capsid amino acids (Grimm et al., 2008). An important characteristic of AAV-DJ capsid is that it was artificially selected with pooled human antisera. As a result, the capsid resistance to neutralizing antibodies against AAV was significantly increased (Grimm et al., 2008).

Preclinical studies on relevant disease model are a prerequisite for developing a safe therapeutic AAV vector. Today, mouse disease models prevail over rat ones, even though in some cases, rat models are preferable. There are several established clinically relevant rat models of disease including myocardial injury, hindlimb ischemia and nerve regeneration. (Mcmahon et al., 2006). Human and rat hearts consist of about 30% myocytes and 70% non-myocytes whereas the mouse heart consists of about 55% myocytes and 45% non-myocytes (Banerjee et al., 2007; Vliegen et al., 1991). The possibility of using the same AAV vector on human and different animal models is an undisputed advantage. Nevertheless, most AAV serotypes appear to be species-specific and have specificity for particular cell types. For instance, AAV serotypes 1,2,4,5,6 are unable to infect rat MSCs (Mcmahon et al., 2006).

To the best of our knowledge, there have been no studies in which rat MSC were transduced with AAV-DJ, although the study on MSC from mouse bone marrow shows that AAV-DJ infects cells five times more efficiently than AAV2 (Lakhan et al., 2015).

The present paper aims to investigate infection efficacy of serotypes AAV-DJ and AAV2 on human and rat adipose tissue-derived MSC and examine the impact of transduction itself on cell viability. Intracellular green fluorescent protein (GFP) and secreted stem cell factor (SCF or KITLG), were selected as transgenes. Rat c-KIT+ cardiac progenitor cells expressing the cognate SCF receptor c-KIT were used to estimate functional activity of transgenic SCF.

## RESULTS

### Efficiency of human adipose tissue-derived MSCs transduction by AAV-2 and AAV-DJ vectors serotypes

**Fig. 1.**
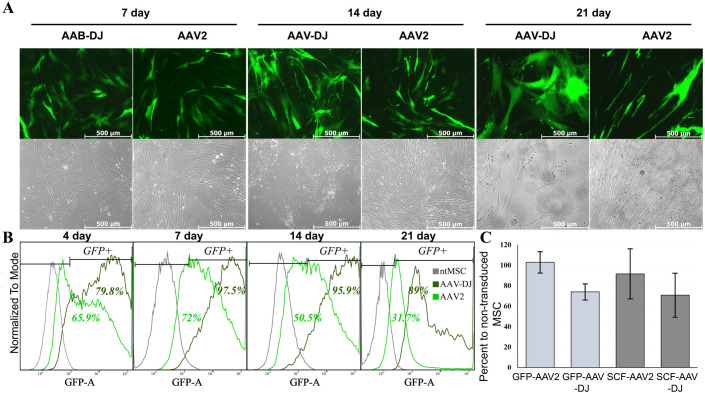
**GFP expression efficiency in human MSCs after transduction with AAV-DJ or AAV-2.** (A) top panels show representative fluorescence photomicrographs of transduced cells (green channel) at 100× magnification; lower panels shows the same field of view in phase contrast. (B) Typical flow cytometry histograms showing GFP expression in human MSCs transduced with AAV2 or AAV-DJ from 4th to 21st day. The percentage of GFP-positive cells in intact MSC (control) or transduced with AAV-DJ showed at the top of the frame. (C) Trypan blue stained MSC were counted at a magnification of ×100 in triplicate. Data are expressed as mean±s.d.

We at first performed optimization of human MSC transduction protocol with new AAV vector serotype AAV DJ. The highest infection efficacy was observed in cells that reached 70-80% confluency (∼2.5×10^6^ cells). Stable expression of the reporter gene (GFP) was detected up to 21 days, by flow cytometry and fluorescence microscopy (Table 1, Fig. 1). We revealed differences about 30% between the two serotypes AAV-2 and AAV-DJ in both the transduction efficacy and the dynamics of the percentage of infected cells over time.

**Table 1.**
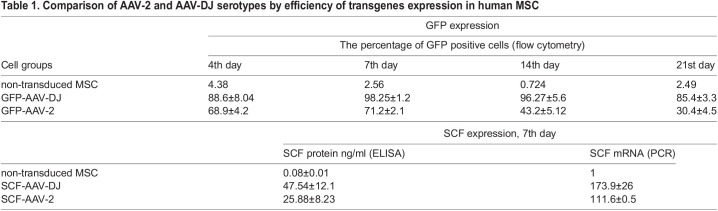
Comparison of AAV-2 and AAV-DJ serotypes by efficiency of transgenes expression in human MSC

Similar results were obtained for vectors expressing secreted transgenes. SCF mRNA expression levels were estimated by qRT-PCR and recombinant protein concentration into culture medium was measured by ELISA.

According to our previous data, the protein secretion level reaches its peak on days 7-9 after AAV transduction (Boldyreva et al., 2019). Table 1 shows ELISA and PCR data for the seventh day after infection. Transgenic SCF mRNA expression was detected with primers specific to codon-optimized SCF (‘huSCF-mod’, Table 2) and its ΔΔCt were compared with ΔΔCt of natural SCF in intact MSC, measured with primer pairs ‘huSCF-natural’ (Table 1). Qualitative mRNA expression data show a good correlation with protein expression estimated by ELISA. By both methods, SCF expression from AAV-DJ was more than 1.5 times higher than AAV-2.

**Table 2.**
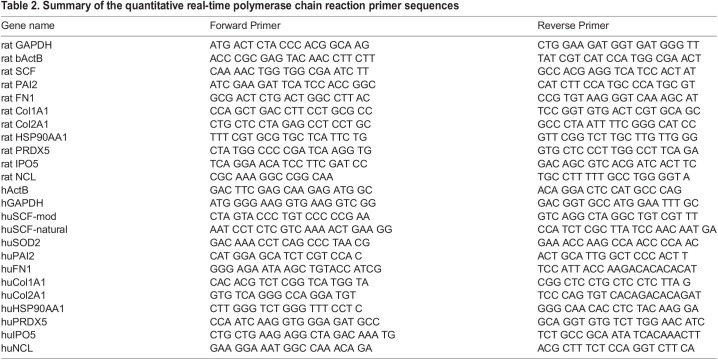
Summary of the quantitative real-time polymerase chain reaction primer sequences

### The effects of viral transduction on MSC proliferation and gene expression profile

Routine cell-counting procedure during sample collection for ELISA and PCR assays revealed the ﻿diminished rate of cell growth in transduced cells. The number of cells in culture of MSC transduced by AAV-DJ was approximately 30% lower than in the intact MSC on days 7-14 after infection (Fig. 1B). We supposed that difference between AAV serotypes is most likely due to the different efficiency of transduction. While AAV-DJ infects 98% of MSC population, only 70% of MSC were transduced using AAV2. The proportion of the uninfected cells increased significantly over time compared to infected ones, due to the difference in the cell division rate. Since the percentage of infected cells in the population was initially lower in AAV-2-MSC than in intact MSC, the lag in cell growth became less apparent over time. This can be clearly seen in Fig. 1A.

﻿It is well documented that AAV infection may elicit DNA-damage-response signaling pathways that results in cell cycle disturbances (Fragkos et al., 2009; Saudan et al., 2000; Winocour et al., 1988), among other responses. ﻿Cell cycle perturbation is dependent upon the p53 activity and its downstream regulatory cascades. Human embryonic stem cells and hiPSC or cancer cells that lack p53 activity undergo AAV-induced apoptosis upon transduction. (Rapti et al., 2015; Hirsch et al., 2011; Raj et al., 2001). This is particularly interesting for us because MSCs are also considered to be progenitors.

We observed minor cell death in human adipose-tissue-derived mesenchymal stromal cells only at the first day after transduction, but a delay in cell proliferation was observed throughout the entire duration of the experiment (21 days).That was particularly noticeable in the case of AAV-DJ.

DNA damage response may be associated with oxidative stress. We hypothesized that infection with AAV vectors could also provoke oxidative stress in MSC. AAV-2 binding to its entry receptors leads to activation of the Rac1-PI3K signaling pathway (Sanlioglu et al., 2004). Small GTPase Rac1 is well known as a key regulator of NADPH-dependent membrane oxidase (NOX), which is one of the most important sources of intracellular reactive oxygen species (ROS) (Ferro et al., 2012).

**Fig. 2.**
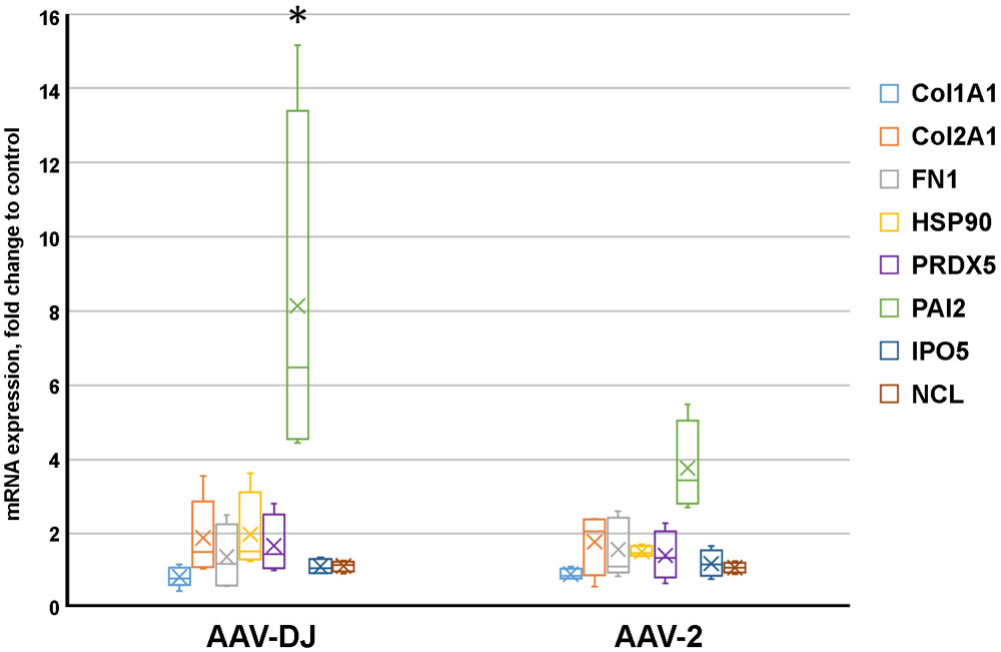
**Comparison of changes in gene expression levels in human MSC after transduction with AAV-DJ or AAV-2.** Box plot showed the normalized relative mRNA expression of nine genes, at 7th day after transduction with AAV-viral vectors assessed by real-time PCR analysis. Values were calculated as ratio to mRNA expression levels in intact MSC. The thick line inside the box plot indicates the median expression levels and the box shows the 25th and 75th percentiles, while the whiskers show the maximum and minimum values. Statistical significance of expression changes in AAV-DJ vs AAV2 was determined using the *t*-test (*P*<0.05).

Based on the our assumption that AAV can provoke oxidative stress in MSC, we analyzed the mRNA expression of two antioxidant enzymes: Peroxiredoxin 5 (PRDX5) and Mn superoxide dismutase (SOD2), SERPINB2 and HSP90 in human MSC after 7 days post infection (Fig. 2).

It was shown that expression of SERPINB2 (also known as PAI-2; type 2 plasminogen activator inhibitor) increases in response to oxidative stress (Cho et al., 2019). PAI-2 supposedly could serve as a biomarker for predicting toxic responses such as defective cell proliferation (Lee et al., 2018). The induction of heat shock protein expression is also one of the main mechanisms to protect cells from damage caused by oxidative and other types of stress (Samali and Orrenius, 1998). Heat shock protein 90 (Hsp90) is the most common chaperone, and accounts for 1-2% of total cellular proteins or up to 4-6% under stress conditions (Bohush et al., 2019). In addition, the heterocomplex of chaperones FKBP52/HSP90 participates in the regulation of AAV transduction. FKBP52 interacts specifically with the D-sequence within the inverted terminal repeat of the AAV genome and forms a complex with HSP90 (Zhong et al., 2004).

We observed statistically significant difference between AAV-DJ and AAV-2 vectors only for PAI-2 expression. The HSP90 mRNA levels tended to increase in MSC transduced by AAV-DJ (Fig. 2A). The expression of PRDX5 did not differ significantly between the serotypes but showed an upward tendency in comparison with intact MSC (Fig. 2A). The expression of another antioxidant enzyme, SOD2, did not change (the data are not shown).

Pro-inflammatory state of MSC leads to changes in collagen or fibronectin deposition (Waterman et al., 2010). Thus, its expression could also serve as an indirect indicator of MSC state, but we did not find significant differences in the expression of these genes between intact MSC or MSC infected with viral vectors.

Finally, AAV use some import proteins for nuclear entry, which is a crucial event in the viral life cycle, since virions that do not enter the nucleus are subjected to proteases or proteasomal degradation by host cells (Li et al., 2013). Previously, we detected increased protein levels of importin and nucleolin in extracellular vesicles from AAV-infected MSCs (Zubkova et al., 2019) but in the present work mRNA levels of importin and nucleolin at day 7 post-infection were unchanged.

With a few exceptions, our results show that at day 7 post-infection there were no significant differences in the expression of selected genes between intact MSC or MSC infected with viral vectors of both serotypes. PAI-1 and HSP-90, which presumably can serve as stress indicators were upregulated, especially in the case of AAV-DJ.

### AAV-DJ vector mediates efficient transduction of rat adipose tissue-derived mesenchymal stromal cells

**Fig. 3.**
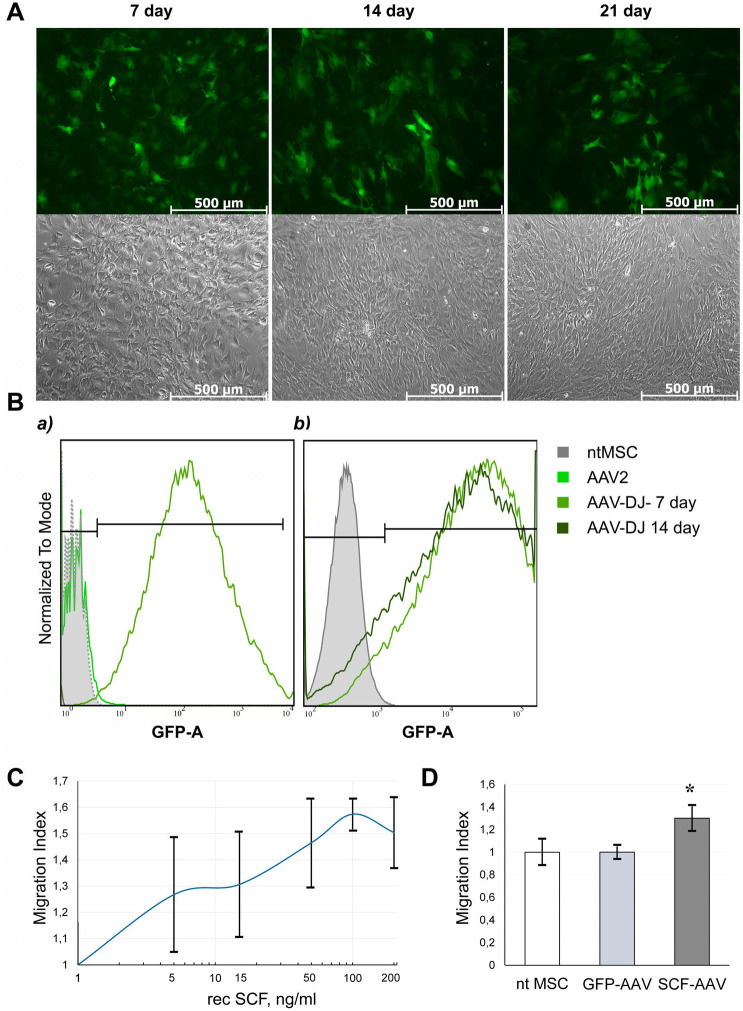
**GFP expression efficiency and SCF functionality in rat MSCs after transduction with AAV-DJ.** (A) AAV-DJ-infected rat MSCs exhibited robust GFP signal. Phase contrast (top) and green channel (bottom) representative images at 100x magnification. (B) Flow cytometry histograms showing GFP expression in rat MSCs transduced with AAV2 or AAV-DJ (a) and changes in GFP expression with AAV-DJ on the 7th and 14th days after infection (b). (C) Transwell migration assay of rat c-kit+ cardiac progenitors. Cells were allowed to migrate toward increasing concentrations of recombinant rat SCF in serum-free medium overnight. Five random fields of view were photographed for each sample, and number of migrated cells per FOV was counted. The cell migration index was calculated as the ratio of cells migrated toward SCF to cells migrated toward assay media. (D) Migration of rat c-kit+ cardiac progenitors toward conditioned media from the intact rat adipose tissue-derived MSC or transduced with GFP-AAV-DJ and SCF-AAV-DJ respectively in Transwell chamber. Data are expressed as the mean migration index±s.d. *P<0.05 versus ntMSC.

The distinctive feature of AAV serotypes is their different tropism for certain tissues and species specificity (Rajapaksha et al., 2019). The transduction efficiency of AAV2 vector in rat adipose-derived mesenchymal stromal cells does not exceed 4% (Fig. 3Ba). These observations were confirmed by other researchers who compared a range of AAV serotypes (1,2,4,5,6) by transduction efficiencies of rat MSC and found that MSC were refractory to transduction by AAVs of all serotypes tested (Mcmahon et al., 2006).

Considering these observations, we attempted to infect rat MSC with DJ serotype using the same vector, GFP-AAV-DJ, that was applied for human MSCs and AAV-DJ vector encoding rat SCF. GFP expression was evaluated by flow cytometry and fluorescence microscopy (Fig. 3). SCF secretion was measured in the supernatant of infected rat MSC by ELISA and expression of SCF mRNA was confirmed by qRT-PCR. Data are shown in Table 3.

**Table 3.**
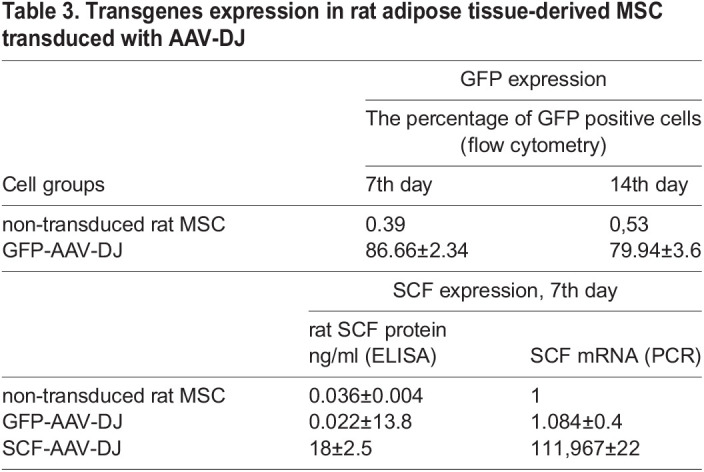
Transgenes expression in rat adipose tissue-derived MSC transduced with AAV-DJ

The novel hybrid vector AAV-DJ showed a superb transduction efficiency in rat MSC: about 80% of GFP+ cells, which is only 10% less than the efficiency in human MSC. SCF expression was measured by both ELISA and PCR data (Table 3).

### AAV DJ provides production of biologically active transgene in rat MSC

Since, to the best of our knowledge, AAV-DJ has not been previously used for the rat adipose tissue-derived MSC transduction, we decided to estimate the functional activity of transgenic SCF (Fig. 3B).

The SCF receptor- c-kit (KITLG) is known to be present in resident cardiac progenitor cells. C-kit+ cells were isolated from the outgrowth cells of the rat heart explants by immunomagnetic selection. Because SCF is an important chemotactic factor for stem and progenitor cells we examined whether SCF secreted by AAV-DJ transduced rat MSC increased c-kit+ cells migration in Transwell™ assay. The conditioned media collected from genetically engineered rat MSC better stimulates migration response of c-kit+ cells compared to the media from intact MSC (Fig. 3). The effect of MSC conditioned media containing 16-18 ng/ml SCF in average corresponded to those observed for recombinant rat SCF (a positive control) at the same concentration (Fig. 3D). These data indicate that transduced rat MSC produce SCF with full functional activity.

### Effect of transduction on the properties of rat adipose-tissue-derived mesenchymal stromal cells

**Fig. 4.**
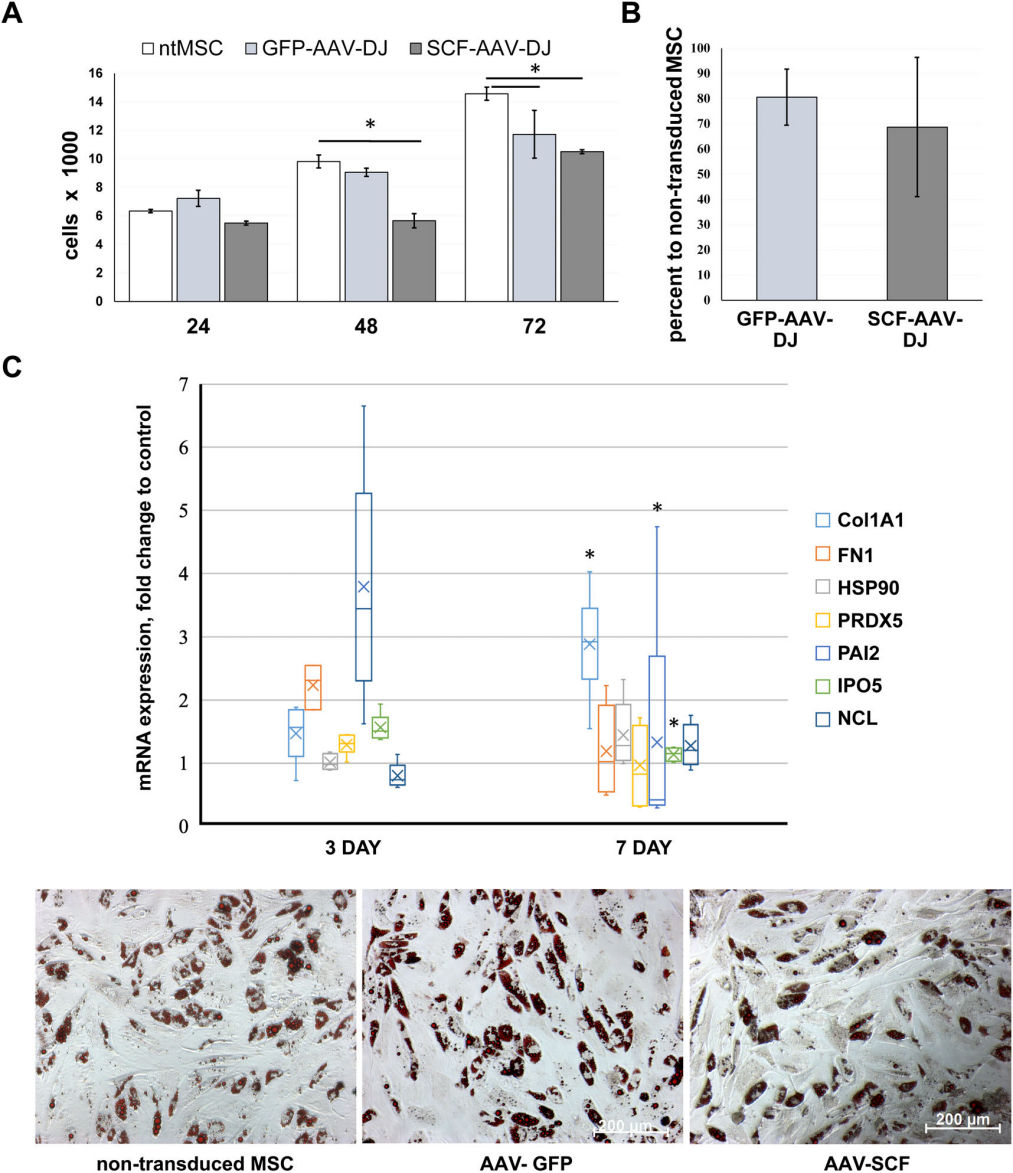
**Influence of transduction with AAV-DJ viral vector on growth rate, mRNA expression profiling, and adipogenic capacity of rat adipose-tissue derived MSCs.** (A) MTT test, data are presented as mean±s.d.. (B) Growth rate slowdown of infected MSC, estimated by manual counting at 7th day after infection; the number of intact cells is taken as 100%. (C) Change in mRNA expression at 3rd and 7th day in infected rat MSC relative to intact cells. The thick line inside the box plot indicates the median expression levels and the box shows the 25th and 75th percentiles, while the whiskers show the maximum and minimum values. Statistical significance (7 day versus 3 day) was determined using the *t*-test (*P*<0.05). (D) Adipogenic differentiation of adipose tissue MSCS after viral transduction. Typical photomicrographs were taken on 21 day after the start of differentiation induction in adipogenic media. Oil red O staining (red color represents the lipid accumulation) was visualized by phase contrast microscopy at 200× magnification. The inverted microscope AxioObserver A1 (Zeiss) was used.

Based on our data obtained for human MSC we assumed that rat MSC proliferation might also be affected by AAV-DJ vector. To determine how transduction can change the MSC proliferation rate we carried out a manual cell counting and MTT assay. Cell viability comparison was performed under standard culture conditions (DMEM, 10% FSB). Transduced cells exhibited significantly slower proliferation rate as compared to intact rat MSC after 48 h post infection (Fig. 4A), but almost all cells were viable.

Results of the cell count analysis was consistent with the MTT assay data (Fig. 4B), in this case observed proliferation rate of genetically modified MSC was 20-30% lower than that of intact MSC.

We evaluated changes in mRNA expression levels of the same stress markers genes whose expression was studied in human MSC by qRT-PCR. The expression of all genes except Col1A1 did not change significantly at day 7 compared to intact cells in contrast to human MSC. This finding was somewhat unexpected because previously we found an increase in the level of some of these proteins (PRDX5, NCL, IPO5) in the content of extracellular vesicles cargo from transduced cells (Zubkova et al., 2019). Considering this, we assumed that changes in mRNA expression in rat MSC most likely occurred ahead of the corresponding changes in the protein levels and therefore estimated mRNA expression at an earlier time point, day 3. The results are presented in Fig. 4C. Expression of a PAI2, Col1A1 and IPO5 on day 7 changes significantly compared to day 3 that allows us to assume the stress response activation in rat MSC after transduction with AAV-DJ.

Lastly, we assessed the potential impact of AAV-DJ transduction on the ability of rat adipose tissue-derived MSC to differentiate toward the adipocytic lineage. After 21 days of incubation in adipogenic induction medium, lipid vacuoles in mature adipocytes was visualized by Oil Red O (Merck Millipore) staining. The analysis of the obtained images revealed no statistically significant differences between infected or intact cells (Fig. 4D).

We show that high levels of transduction achieved using the DJ vector have no inhibitory effect on the ability of rat MSCs to differentiate down the adipogenic cell lineage.

## DISCUSSION

Both gene and cellular therapy offer perspective strategies for effective treatment of inherited and acquired diseases. However, the combination of these methods reinforces their potency and allows specialists to use the combined therapeutic possibilities.

To date, gene therapy with adeno-associated viral vectors is becoming an innovative platform for the treatment of persistent congenital or acquired heart diseases. ААV viruses do not provoke human diseases and provide long-term extrachromosomal episome based transgene expression without the genome integration (Chamberlain et al., 2017).

At the initial stage of our study, we compared two serotypes: the most used serotype AAV-2 and a hybrid serotype AAV-DJ created based on natural serotypes AAV2/8/9. Vectors were matched by the efficiency of transduction (GFP) and the level of transgene expression (SCF) in human MSC. In all cases, the efficiency of infection with the AAV-DJ was higher than with AAV-2 and approached 100%. The percentage of GFP-positive cells remained high up to 21 days.

Stem cell factor gene (SCF) was used as the second (secreted) transgene. This chemokine is attractive candidate for myocardium gene therapy. It was previously demonstrated that transgenic SCF improve survival and cardiac function in a rat model of myocardial infarction (Yaniz-Galende et al., 2012). Both ELISA and PCR data demonstrated that SCF expression was about 1.5 times higher with AAV-DJ serotype than with AAV2.

We also noticed a remarkable slowdown in the growth of transduced cells regardless of serotype used. This effect was more significant for AAV-DJ, which is due to the lower percentage of non-transduced cells in the population. This phenomenon was reported earlier for parvovirus family, to which AAV belongs (Winocour et al., 1988; Hermanns et al., 1997; Morita et al., 2001). Recombinant AAV-based vectors can also cause alterations in the proliferation or even induce apoptosis depending on the cell type and activity of a signaling cascade p53-p21-pRb (Hirsch et al., 2011). It is suggested that the DNA damage response is provoked by inverted terminal repeats (ITR) which are present in both the wild-type and recombinant vectors (Raj et al., 2001).

Besides, we revealed an increase in mRNA levels of plasminogen activator inhibitor type 2 (PAI-2, also known as SERPINB2) in AAV-transduced human MSC. PAI-2, along with PAI-1, inhibits plasminogen activation, by covalent binding to proteolytic domains of plasminogen activators, urokinase or tissue-type-plasminogen activator (Medcalf, 2011). In contrast to PAI-1, PAI-2 exhibits intracellular activity. A significant upregulation of PAI-2 expression was reported previously in response to DNA damage, replicative senescence and oxidation stress (Cho et al., 2019) or cytotoxic agents (Lee et al., 2018). Thus, PAI-2 might be considered a common stress response protein (Hsieh et al., 2017). On the other hand, PAI-2 can induce growth arrest via p21 stabilization to help cells accommodate stress (Hsieh et al., 2017). In our case, persistent PAI-2 up-regulation on the seventh day after transduction (3-6 times higher than observed in intact cells) may indicate a prolonged stress process. The expression of another stress-response factor (HSP90) showed a tendency to an increase only in MSC transduced with AAV-DJ.

Of particular importance in our study is the use of the AAV-DJ vector for transduction of rat adipose-derived mesenchymal cells.

Today, mouse disease models prevail over rat ones, even though in some cases, rat models are preferable. Bigger size and weight of rats provide some practical advantages, especially in surgery, where rat models show more translational value. Besides, mouse mortality associated with myocardial infarction induction is about 37-50%, while the mortality of Sprague-Dawley rats was 36%, whereas in Lewis inbred rats it was about 16% (Klocke et al., 2007). There are specific physiological differences between the rat and mouse, such as heart rate, total collagen and myocardial cell composition (Banerjee et al., 2007; Vliegen et al., 1991). Therefore, the possibility of using rat model for preclinical studies with genetic-therapeutic vector is an undisputed advantage. However, AAV serotype vectors significantly vary by tissue tropism (Srivastava, 2016) and majority of AAV vectors infect rat MSC quite inefficiently (Mcmahon et al., 2006), which we showed with AAV2.

Here we demonstrate that AAV-DJ can transduce rat adipose-tissue-derived MSC with an efficiency comparable to human MSC. Transgenes mRNA expression levels were slightly lower in rat MSC by 10% for GFP and 20% for SCF. Cells growth of the infected rat MSC was reduced by 20-30% compared to intact cells, which was similar for human adipose-tissue-derived MSC.

Further analysis revealed differences in the response of rat and human MSC to transduction: the expression level of PAI-2 mRNA increased in rat MSC at an earlier time point than in humans.

As we showed earlier, transduction with AAV vector did not affect the differentiation potential of human adipose-tissue-derived MSC (Shevchenko et al., 2013). This study has confirmed our previous findings. We verified that AAV do not affect adipogenic capacity in rat adipose-tissue-derived MSC as well.

Functional activity of transgenic SCF was estimated on rat c-kit+ cardiac progenitor cells expressing cognate SCF. Such analysis was not performed on human cells due to the extremely limited availability of human myocardium. Exposure to SCF-containing MSC conditioned media enhanced migration of c-kit+ cardiac progenitor cells and correlated with data obtained with recombinant rat SCF in the same concentration. This suggests that AAV-expressed SCF retain chemotactic properties.

In summary, we showed that AAV-DJ, unlike AAV-2, transduced human and rat MSC with comparable efficiency and surpassed AAV-2 in infection efficiency. Transgene expression lasted for at least 21 days (termination point of the study). The infected MSC still viable and preserves adipogenic differentiation capacity but infection with AAV viral vectors led to a cell growth slowdown in both rat and human MSC. These findings are important for further use of genetically modified cells secreting SCF or other proteins with promising therapeutic potential in pre-clinical and clinical applications. Furthermore, our data expand on previous studies by demonstrating that AAV infection itself has an important effect on MSC behavior, including growth rate slowdown and expression of the stress factor PAI2. We revealed that there is a differential cellular response in MSCs transduced with AAV viral vectors, and that these changes may affect an important MSC function and should be taken into account since they can impact the expected therapeutic effect. Elucidating the full repertoire of AAV-elicited signaling in MSC will be important in understanding the capacities of genetically modified MSCs in cellular therapy.

## MATERIALS AND METHODS

### Isolation and culture of adipose-tissue-derived mesenchymal stromal cells

Adipose-tissue-derived human mesenchymal stromal cells were isolated from the subcutaneous adipose tissue of patients aged 21-55 years who underwent biopsies. It should be especially noted that adipose tissue was taken exclusively from male patients. It is necessary because for AAV2 the gender specificity of transgene expression was shown, at least for liver tissue. Thus, the efficacy of liver infection in male mice by AAV2 vector *in vivo* was approximately seven times higher than that of female mice (Davidoff et al., 2003).

All donors have given informed consent to experimental manipulations of adipose tissue samples. Donors with infectious, systemic diseases or malignancies were not included in the study. In order to isolate MSC, pieces of subcutaneous adipose tissue were minced to a size of 1-2 mm and digested with collagenase I (200 units/ml) (Sigma-Aldrich, USA) and dyspases (30 units/ml) (Thermo Fisher Scientific Inc., USA) solution for 1 h at 37°C. Centrifugation (200 ***g***, 10 min) yielded precipitated cells which were resuspended and filtered through a 40 µm nylon cell strainers (BD Bioscience, USA). The filtered suspension was again centrifuged (200 ***g***, 10 min). Cell pellet was resuspended in a complete growth medium -DMEM-F12/10% FBC (Gibco, USA) and cultivated under standard conditions (5% CO2, 37°C). The next day, the unattached cells and red blood cells were washed out and the medium was replaced with a fresh one. Then the medium was changed every 2-3 days. Cells were passaged upon reaching 70% confluency using 0.05% trypsin/0.02% EDTA dissociating solution (PanEco, Russia) and subcultured at a 1:4 ratio. Cells were used up to and including passage 4.

Rat adipose-tissue-derived mesenchymal stromal cells were isolated from the subcutaneous adipose tissue of Wistar-Kyoto adult males at 12-24 weeks of age. Surgical manipulations and euthanasia protocols were designed in accordance to Institute and National regulations. All animal experiments were carried out in compliance with internal ‘Rules for conducting work using experimental animals’ and were approved by the Ethical Board of the National Medical Research Center of Cardiology. The isolation was performed as described above for human mesenchymal stromal cells.

### Isolation of c-kit +precursor cells from rat myocardium

C-kit+cells were isolated following the protocol we developed earlier (Dergilev et al., 2018). Briefly: hearts of Wistar male rats (250-300 g) were digested 15 min in a mixture of 0.1% collagenase (Roche Diagnostics, USA) and of 0.2% trypsin (Invitrogen, USA). After enzymatic treatment, pieces of myocardium were cultivated as explants for 10 days in DMEM/F12/10% FBS/10 ng/ml LIF culture medium. Then cells expressing the SCF receptor c-kit were separated by magnetic-activated cell sorting using the Goat Anti-Rabbit IgG MicroBeads (Miltenyi Biotec, USA) coated with rabbit c-kit antibody (H-300) (sc-5535 Santa Cruz Biotechnology, Inc., USA) according to beads manufacturer protocol (Miltenyi Biotec, USA).

Isolated c-kit+cells were grown on fibronectin-coated Petri dishes and maintained in DMEM/F12/10% FBS medium supplemented with 2% B27, insulin-transferrin-selenium (ITS), bFGF (20 ng/ml), EGF (20 ng/ml) and LIF (10 ng/ml).

All institutional and national guidelines for the care and use of laboratory animals were followed.

### Proliferation of adipose-tissue-derived mesenchymal stromal cells

The viability of rat MSC was assessed by manual counts with trypan blue exclusion stain and by MTT assay (Mosmann, 1983). In brief, cells (transduced and intact) were seeded on three 96-well plates at 5000 cells per well in a final volume of 0.1 ml. To generate a calibration curve required to convert absorption measurements into equivalent cell counts, serial dilutions of MSC (from 620 to 20,000 cells per well) were seeded in the same 96-well plates. Cells were incubated for 24, 48 and 72 in complete growth medium. At the end of incubation time cell proliferation was assessed by directly adding 20 µl the solution of MTT [3-(4,5-dimethyltiazole-2-il)-2,5-diphenyltetrazolybromide] (2.5 mg/ml) to culture medium for another 4 h. Then the medium was completely removed and the precipitated formazan crystals were dissolved in acidic isopropyl alcohol. The absorbance was read on a Multiscan Microplate Reader (Labsystems, USA) using a test wavelength of 595 nm, a reference wavelength of 630 nm. The number of cells per well (thousands) was calculated using a calibration curve.

### Quantitative reverse transcriptase PCR (qRT-PCR)

Total RNA was extracted using an RNeasy Mini Kit (Qiagen, USA) according to the manufacturer’s protocol. RNA concentration was measured using a NanoDrop 2000 spectrophotometer (Thermo Fisher, USA). First strand cDNA was synthesized from one microgram of total RNA using the High Capacity cDNA Reverse Transcription Kit (Thermo Fisher, USA) following the manufacturer's protocol. qRT-PCR was performed on a StepOnePlus™ Real-Time PCR System (Applied Biosystems, USA). The reaction mixture contained a SYBR Green PCR Master Mix (Syntol, Russia), primers (10 pmol of each dNTP) and cDNA (1-5 ng cDNA). After the initial stage of denaturation (95°, 10 min) 40 amplification cycles of 95°C for 15 s and 60°C for 60 s were performed followed by melt curve analysis. The primer sequences (Eurogen, Russia) are shown in Table 2. mRNA levels between probes were normalized by the mRNA levels of beta-actin (bActB) and glycerylaldehyde-3-phosphate dehydrogenase (GAPDH) as a housekeeping genes and the 2−ΔΔCt method was used to calculate the relative expression levels of genes.

### Adipogenic differentiation

The effect of transduction on the MSC ability to adipogenic differentiation was tested using the Mesenchуmal Stem Cell Adipogenesis Kit (Millipore, USA) according a manufacturer's protocol. MSC were seeded into 24-well plates at a density of 60×10^3^ cells per well. Cultures were re-fed every 2 days for 21 days with a complete change of adipogenic medium. Histological dye Oil Red O (Millipore, USA) was used to visualize the accumulation of lipid droplets in cells. Cells cultivated in medium without adipogenic supplements were used as negative control.

### Transduction of MSC by AAV

A codon-optimized human SCF gene or a rat's natural SCF gene were cloned in VPK-410 expression vector (Cell Biolabs, Inc., USA). The ready-made GFF-expressing vector AAV-400 (Cell Biolabs, Inc., USA) was used for control. Serotype-specific packaging vectors: VPK-420-DJ (ААВ- DJ) or VPK-422 (ААВ-2) and pHelper Vector (number 340202) (Cell Biolabs, Inc., USA) together with expression plasmid were used to co-transfect packaging cells (HEK293T) using the calcium phosphate transfection method, to obtain AAV vector particles with different capsid proteins. 48 h post-transfection recombinant adeno-associated viruses were collected according to the instructions of the manufacturer (Cell Biolabs, Inc., USA).

Mesenchymal cells were cultured in 100 mm2 dishes until they reached 80% confluency. Afterwards they were infected with viral vectors of AAV2 or AAV-DJ serotypes (stock 5.5×10^10^ gc/ml, MOI 100000). Cells were incubated for 3 h with mild stirring every 30 min. Afterwards, 5 ml of DMEM/F12 medium supplemented with 20% FBS was added and MSC were cultivated overnight, then half of the culture medium was replaced with a fresh one. Cells were passaged 3 days after infection and samples for qRT-PCR or ELISA were collected on the 7th day.

### ELISA assay

Intact and transduced MSC were cultivated in six-well plates or in a 60 mm^2^ Petri dishes in a complete growth medium (DMEM/10% FBS) for 48 h. The conditioned media were collected, centrifuged to remove floating cells (400 ***g***) and debris (2000 ***g***) and stored at −70°C before analysis. SCF concentrations were measured using Human SCF Quantikine ELISA Kit or SCF Mouse ELISA Kit (Abcam, USA) following to the manufacturer’s instructions. Optical density in 96-well strip plates was measured in Victor™ X3 Multi Label Plate Reader (Perkin Elmer Inc, USA).

### Flow cytometry

For flow–cytometric analysis, MSC infected with GFP-AAV-DJ or GFP-AAВ-2 were trypsinized (0.05% trypsin) and rinsed twice with PBS. GFP expression was measured by FACSCanto™ II flow cytometer (Becton Dickinson, USA). A total of 1×10^4^ events were analysed for each sample. Data were analyzed using FlowJo™ 10 software (BD, USA).

### Transwell™ migration assay

Transwell^®^ Permeable Supports (BD Falcon, USA) were used to assess the ability of transgenic SCF to induce directed migration of rat cardiac c-kit+ cells. Migration assay was performed using 24-well Transwell^®^ inserts (8 μm) coated with 100 µg/ml rat collagen I solution (IMTEK) at 37°С for 1 h. C-kit+ cells were serum-deprived overnight then harvested by 2 mM EDTA/PBS solution and centrifuged at 200 ***g*** for 10 min. The pellets were resuspended in a chemotaxis medium (DMEM/0.25% FBS) at a concentration of 400,000 cells/ml or 20,000 cells/well and added into the Transwell^®^ inserts that were subsequently placed into the 24-well plate filled with assay media, conditioned media from transduced or intact MSC or recombinant SCF solution in assay media. Cells were allowed to migrate for 20 h, after which cells inside transwell inserts were removed with a cotton swab. Cells that migrated were fixed for 10 min in cold 70% ethanol and stained with hematoxylin-eosin for 30 min and subsequently washed thoroughly with tap water. Images were captured using an inverted microscope AxioObserver A1 (Zeiss, Germany) (magnification, ×200). The number of migrated cells in five randomly selected fields of view was calculated using the ImageJ software (NIH, USA). The results were expressed as migration index defined as: the average number of cells per field for recombinant SCF/the average number of cells per field for the medium control and as the average number of cells per field for transduced MSC-media/the average number of cells per field for the non-transduced MSC-media.

### Statistical analysis

Statistical analysis was undertaken using a paired Student's *t*-test for migration and unpaired two-tailed test for PCR data. *P*-values that were ≤0.05 were considered to be statistically significant. Data are presented as means of three independent experiments (with two or three technical replicates)±standard deviation.
